# Optimizing drug selection in psychopharmacology based on 40 significant CYP2C19- and CYP2D6-biased adverse drug reactions of selective serotonin reuptake inhibitors

**DOI:** 10.7717/peerj.7860

**Published:** 2019-10-09

**Authors:** Andy R. Eugene

**Affiliations:** Independent Neurophysiology Unit, Department of Psychiatry, Medical University of Lublin, Lublin, Poland; Independent Researcher, Kansas, USA

**Keywords:** Pharmacogenomics in psychiatry, Psychopharmacology, Antidepressants side effects, Pharmacovigilance, Precision medicine, Clinical pharmacology, CYP2C19, CYP2D6, Adverse drug reactions, Data science

## Abstract

**Background:**

Selective serotonin reuptake inhibitors (SSRIs) are among the most widely prescribed class of drugs in the practice of psychiatry. Cytochrome P450 (CYP) 2C19 and CYP2D6 are established as clinically relevant drug metabolizing enzymes (DMEs) that influence the pharmacokinetics of SSRIs and may either be grouped as being primarily metabolized by CYP2C19 or CYP2D6. The aim of this study is to test the hypothesis that the primary drug metabolizing pathway for SSRI antidepressants are associated with adverse drug reactions (ADRs) related to physiological modulation of organs with the highest gene tissue expression.

**Methods:**

Post-marketing ADR cases were obtained from the United States Food and Drug Administration’s Adverse Events Reporting System from each of the four quarters for the years 2016 and 2017. Cases were grouped based on one of two primary pharmacokinetic pharmacogenomic pathway biomarkers CYP2C19 and CYP2D6. Citalopram, escitalopram, and sertraline were grouped as CYP2C19 substrates and fluvoxamine, fluoxetine, and paroxetine as CYP2D6 substrates. Logistic regression was computed for the reported SSRI ADRs associated with one of two aforementioned DMEs. All data homogenization and computations were performed in R for statistical programming.

**Results:**

The most commonly reported ADR among the SSRIs was anxiety (*n* = 3,332). The top two ADRs associated with SSRIs metabolized by CYP2D6 are: nightmare (*n* = 983) reporting odds-ratio (OR) = 4.37 (95% confidence interval (CI) [3.67–5.20]) and panic attack (*n* = 1,243) OR = 2.43 (95% CI [2.11–2.79]). Contrastingly, the top two ADRs for CYP2C19 metabolized SSRIs are: electrocardiogram QT prolonged (*n* = 351) OR = 0.18 (95% CI [0.13–0.24]) and small for dates baby (*n* = 306) OR = 0.19 (95% CI [0.14–0.26]). The study tested and produced 40 statistically significant CYP2C19- and CYP2D6-biased ADRs. In overall context, the results suggest that CYPC19 SSRI substrates are associated with ADRs related to modulation of the autonomic nervous system, seizure, pain, erectile-dysfunction, and absorption. Contrastingly, CYP2D6 SSRI substrates are associated with ADRs related to nightmares, withdrawal syndrome, and de-realization of cognitive processes. The results of this study may aid as guidance to optimize drug selection in psychopharmacology.

## Introduction

Selective serotonin reuptake inhibitors (SSRIs) may be dichotomized as clinically relevant substrates of either cytochrome P450 (CYP) 2C19 or CYP2D6 (Hefner, 2018). CYP2C19 and CYP2D6 are known to be highly polymorphic resulting in variation in plasma drug concentrations and are located at cytogenic bands 10q23.33 and 22q13.2, respectively (Zanger & Schwab, 2013). Like most xenobiotics, SSRI antidepressants require metabolic contributions from multiple CYP enzymes and some SSRIs have contributions from both CYP2D6 as well as CYP2C19 (Fermier et al., 2018). However, based on clinically relevant pharmacokinetic parameters such as the area under the concentration-time curve (AUC) and maximum plasma concentration (Cmax), SSRIs then become associated with only one of two isoenzymes CYP2D6 and CYP2C19. Further, in cases where both CYP2C19 and CYP2D6 are evident, the resulting metabolite compounds are known to have weaker transporter and receptor binding affinities, as compared to the parent compound. Moreover, the metabolites are often not clinically relevant when considering exposure-response analysis based on AUC and Cmax. In cases where the parent drug and metabolite compound are both CYP2C19 and CYP2D6 substrates of interest, as in the racemic mixture of *R*- and *S*-fluoxetine, summation of the parent compound and metabolite are reported in therapeutic drug monitoring (Hefner, 2018).

From a regulatory and large-scale population outcomes perspective, the United States Food and Drug Administration (FDA) Table of Pharmacogenomic Biomarkers in Drug Labeling establishes that CYP2D6 is the primary drug metabolizing enzyme (DMEs) for fluoxetine, fluvoxamine, and paroxetine (U.S. Food & Drug Administration, 2016). The Clinical Pharmacogenetics Implementation Consortium as well as the Dutch Pharmacogenetics Working Group guidance documents establish that SSRIs citalopram, escitalopram, and sertraline as metabolized by CYP2C19 (Bank et al., 2018; Hicks et al., 2015). Lastly, the Consensus Guidelines for Therapeutic Drug Monitoring in Neuropsychopharmacology further corroborate the aforementioned SSRI drug-gene pairs, but adds CYP2C9 and CYP2C19 for fluoxetine (Hefner, 2018). With international efforts underway to integrate pharmacogenomic testing to improve both drug- and dose-selection, are we able to predict ADRs of xenobiotics by evaluating gene expression signals across human tissues that most abundantly express DMEs of interest?

**Figure 1:**
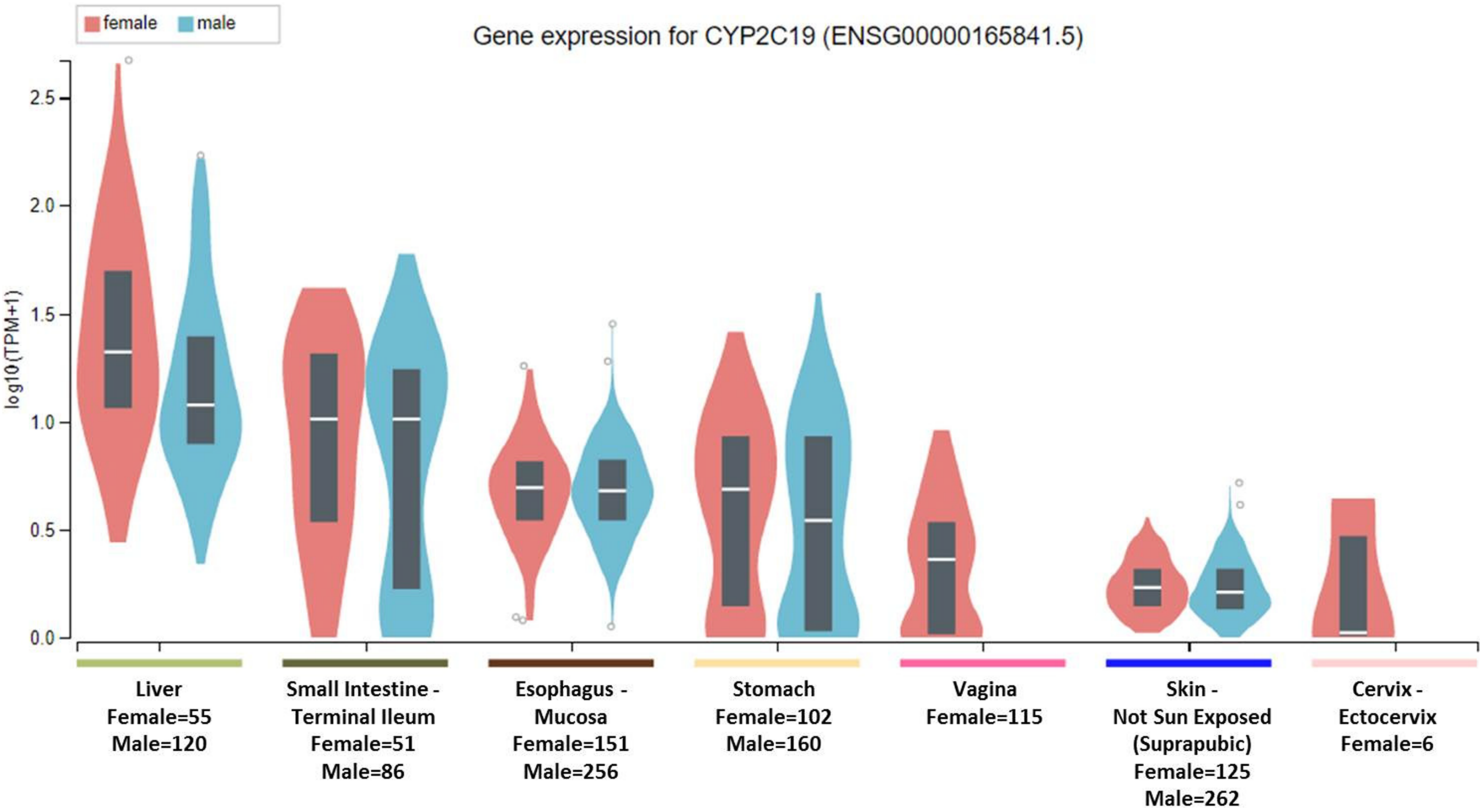
CYP2C19 gene expression across human tissues. The Genotype-Tissue Expression database image for CYP2C19 used to generate the first hypothesis showing gender stratified anatomical structures and sample-sizes on the *x*-axis. The *y*-axis shows the gene expression value in median transcripts per kilobase million (TPM). The boxplot results are sorted based on the median, highest to lowest, gene expression values shown as the horizontal line and dots illustrate population outliers.

**Figure 2:**
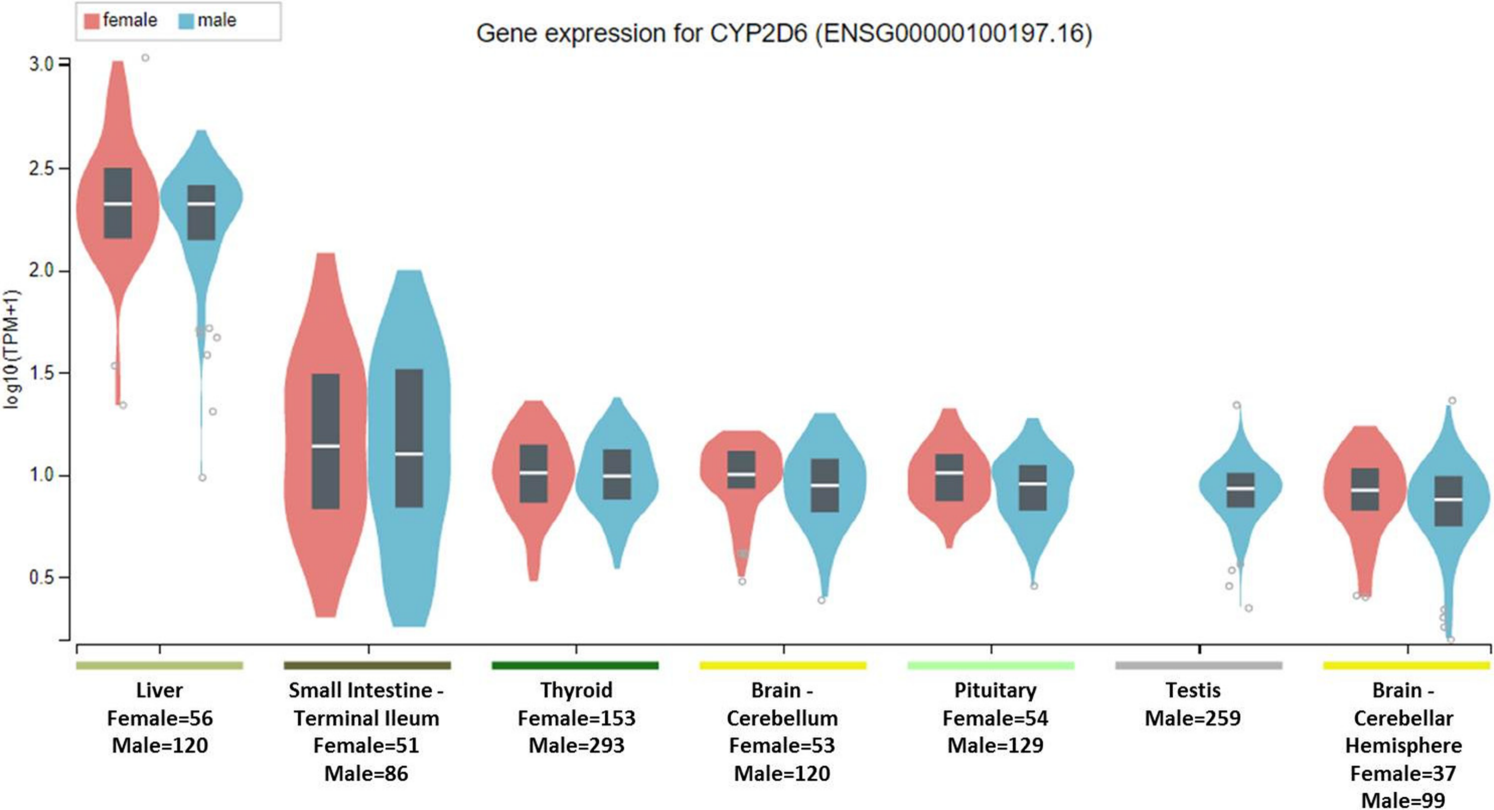
CYP2D6 gene expression across human tissues. The Genotype-Tissue Expression database image for CYP2D6 used to generate the second hypothesis showing gender stratified anatomical structures and sample sizes on the *x*-axis. The *y*-axis shows the gene expression value in median transcripts per kilobase million (TPM). The boxplot results are sorted based on the median, highest to lowest, gene expression values shown as the horizontal line and dots illustrate population outliers.

To clarify the question, it is important to note that the National Institute of Health (NIH) Common Fund sponsored Genotype-tissue expression (GTEx) project database (https://gtexportal.org) reports that the highest gene tissue expression for CYP2C19 and CYP2D6, out of 53 reported anatomical structures, are the liver and terminal ileum of the small intestine (GTEx Consortium, 2017). However, for CYP2C19, following the RNA transcript abundance being in the liver (median transcripts per kilobase million (TPM)-female = 19.88; median TPM-male = 11.01) and terminal ileum (median TPM-female = 9.31; median TPM-male = 9.26), the highest gen tissue expression are subsequently seen in the esophageal mucosa (median TPM-female = 3.96; median TPM-male = 3.79), stomach (median TPM-female = 3.83; median TPM-male = 2.50), vaginal-tissue (median TPM-female = 1.29), suprapubic skin (median TPM-female = 0.71; median TPM-male = 0.61), and the ectocervix (median TMP = 0.05, actually ranked 13 of 56 in anatomic structures), as shown in Fig. 1 (GTEx Consortium, 2017). For the CYP2D6 gene, after the highest expression being in the liver (median TPM-female = 208.4; median TPM-male = 206.9) and terminal ileum (median TPM-female = 12.90; median TPM-male = 11.64), the subsequent highest transcript abundance are found in the thyroid (median TPM-female = 9.27; median TPM-male = 8.79), cerebellum (median TPM-female = 8.97; median TPM-male = 7.91) within the brain, pituitary (median TPM-female = 9.20; median TPM-male = 7.97) within the brain, testis (median TPM-male = 7.54), and cerebellar hemispheres (median TPM-female = 7.39; median TPM-male = 6.58) also within the brain, as shown in Fig. 2 (GTEx Consortium, 2017).

Selective serotonin reuptake inhibitors are widely prescribed among most medical specialties and are indicated in a variety of clinical conditions (Wong et al., 2016). In medical practice, ADRs are a major cause of medication non-compliance and thus precision medicine efforts are helping to alleviate ADRs among patients by using drug pharmacokinetics and pharmacodynamics (PK/PD) (Garon et al., 2017). As an extension of PK/PD principles in clinical pharmacology, pharmacogenomic testing aims to identify which patients lack the ability to metabolize particular drugs (i.e., poor metabolizer) resulting in toxicity, over-metabolize a drug (i.e., ultra-rapid metabolizer) resulting in either sub-therapeutic plasma concentrations or even into toxic metabolite blood levels (e.g., prodrugs as when codeine is metabolized to morphine) (Gasche et al., 2004; Zanger & Schwab, 2013). However, even in patients with normal ranges of blood levels identified via therapeutic drug monitoring or having a phenotype label of being a normal metabolizer—that is by having two functional alleles of the cytochrome P450 enzymes—certain ADRs remain a clinical conundrum (Hefner, 2018; Weinshilboum, 2003).

With this information as a background, the aim of this study is to test the hypotheses that: (1) the CYP2C19-related SSRI ADRs are significantly associated with *gastrointestinal*, *nutritional*, *enteric nervous system*, or *pregnancy-reproduction* related ADRs while, (2) the top two CYP2D6-related SSRI ADRs are significantly associated with physiological effects of *thyroid, pituitary* modulation, and de-realization of *brain* thought processes.

## Materials and Methods

Post-marketing patient cases were identified from the United States FDA Adverse Events Reporting System (FAERS) and downloaded for each of the four quarters of the years 2016 and 2017. SSRIs were categorized based one of two primary DMEs, CYP2C19 or CYP2D6 (Hicks et al., 2015; U.S. Food & Drug Administration, 2016). The following six SSRIs were extracted from the FAERS database and associated with the primary metabolizing pharmacokinetic gene: escitalopram (CYP2C19), citalopram (CYP2C19), sertraline (CYP2C19), fluoxetine (CYP2D6), fluvoxamine (CYP2D6), and paroxetine (CYP2D6). The case-control method was used and tested for ADRs using logistic regression by clustering SSRI substrates of CYP2C19 and CYP2D6 with labels of 0 and 1, respectively. Thus, results odds-ratios (OR) closer to 0 would suggest a CYP2C19-biased ADR and OR greater than 1 suggests a CYP2D6-biased ADR.

During logistic regression, ADR cases were compared to the most common ADR (anxiety, *n* = 3,332) among all SSRIs and results are provided as reporting OR. A detailed overview of all data homogenization and statistics may be referenced in a previous study (Eugene & Eugene, 2018). All analyses were conducted in R (version 3.5.0; R Foundation for Statistical Computing, Vienna, Austria) (R Development Core Team, 2015). A *p*-value of less than 0.05 was considered statistically significant.

## Results

A total of 2,870 cases were identified as associated with SSRIs metabolized by the CYP2D6 enzyme and 2,403 reported ADRs with SSRIs primarily metabolized by CYP2C19. The most commonly reported SSRI side effect was anxiety (*n* = 3,332) and this ADR was established as the control variable when testing specific ADRs for all comparisons. Further, suicidal ideation (*n* = 2,841) was the second most report ADR among all reported SSRI-drug related cases. Neither of the following ADRs were biased toward either DME: mania (*n* = 375) reporting OR = 1.15 (95% confidence interval (CI) [0.93–1.42]), dizziness (*n* = 2,422) OR = 1.09 (95% CI [0.98–1.21]), depression (*n* = 2,808) OR = 1.09 (95% CI [0.99–1.21]), decreased appetite (*n* = 1,348) OR = 0.97 (95% CI [0.85–1.10]), and insomnia (*n* = 1,933) OR = 0.91 (95% CI [0.82–1.02]).

**Figure 3:**
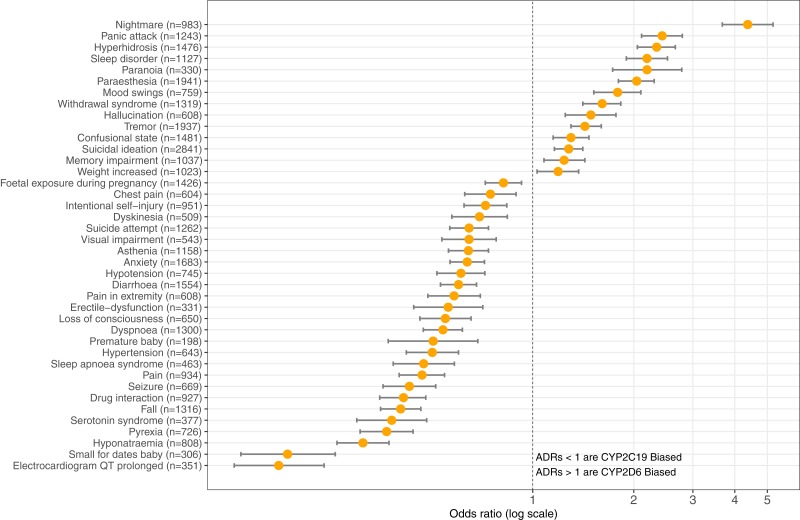
CYP2C19-biased vs. CYP2D6-biased SSRI Adverse Drug Reactions. Log-transformed odds-ratios of 40 significantly associated adverse drug reactions with CYP2C19-SSRI-substrate (less than 1) vs. CYP2D6-SSRI-substrate (greater than 1) selective serotonin reuptake inhibitor class antidepressants.

The top three ADRs associated with SSRIs metabolized by CYP2D6 were found to be: nightmare (*n* = 983) reporting OR = 4.37 (95% CI [3.67–5.20], *p* = 8.67E-62), panic attack (*n* = 1,243) OR = 2.43 (95% CI [2.11–2.79], *p* = 2.94E-36), and hyperhidrosis (*n* = 1,476) OR = 2.34 (95% CI [2.05–2.66], *p* = 5.33E-38). Contrastingly, SSRI antidepressants metabolized by CYP2C19 are significantly associated with the following ADRs: electrocardiogram QT prolonged (*n* = 351) OR = 0.18 (95% CI [0.13–0.24], *p* = 2.88E-28), small for dates baby (*n* = 306) OR = 0.19 (95% CI [0.14–0.26], *p* = 2.52E-24), and hyponatraemia (*n* = 808) OR = 0.31 (95% CI [0.26–0.37], *p* = 2.61E-37). Table 1 provides the OR results for statistically significant comparisons among SSRIs and genes. Figure 3 illustrates the graphical view of log-transformed OR associating CYP2C19 vs. CYP2D6 SSRI medications to ADRs tested in this study.

**Table 1:** Results of 40 statistically significant selective serotonin reuptake inhibitor (SSRI) associated adverse drug reactions, linked CYP2D6 (*n* = 2,870) vs. CYP2C19 (*n* = 2,403) drug metabolizing enzyme, and resulting odds-ratios with 95% confidence intervals.

Adverse drug reaction	Biased gene in SSRI metabolism	Odds-ratio (95% CI)	*p*-value
Nightmare (*n* = 983)	CYP2D6	4.37 [3.670–5.20]	8.67E-62
Panic attack (*n* = 1,243)	CYP2D6	2.43 [2.110–2.79]	2.94E-36
Hyperhidrosis (*n* = 1,476)	CYP2D6	2.34 [2.050–2.66]	5.33E-38
Sleep disorder (*n* = 1,127)	CYP2D6	2.19 [1.90–2.52]	2.41E-27
Paranoia (*n* = 330)	CYP2D6	2.19 [1.73–2.78]	1.28E-10
Paraesthesia (*n* = 1,941)	CYP2D6	2.04 [1.8–2.3]	1.02E-33
Mood swings (*n* = 759)	CYP2D6	1.79 [1.520–2.10]	1.91E-12
Withdrawal syndrome (*n* = 1,319)	CYP2D6	1.61 [1.410–1.83]	5.55E-13
Hallucination (*n* = 608)	CYP2D6	1.49 [1.250–1.77]	8.69E-06
Tremor (*n* = 1,937)	CYP2D6	1.43 [1.3–1.6]	4.00E-10
Confusional state (*n* = 1,481)	CYP2D6	1.3 [1.150–1.47]	3.13E-05
Suicidal ideation (*n* = 2,841)	CYP2D6	1.28 [1.160–1.41]	1.44E-06
Memory impairment (*n* = 1,037)	CYP2D6	1.24 [1.080–1.43]	0.00211
Weight increased (*n* = 1,023)	CYP2D6	1.19 [1.030–1.37]	0.0151
Foetal exposure during pregnancy (*n* = 1,426)	CYP2C19	0.818 [0.722–0.926]	0.00158
Chest pain (*n* = 604)	CYP2C19	0.748 [0.627–0.892]	0.00122
Intentional self-injury (*n* = 951)	CYP2C19	0.723 [0.624–0.837]	1.38E-05
Dyskinesia (*n* = 509)	CYP2C19	0.694 [0.574–0.84]	0.000173
Suicide attempt (*n* = 1,262)	CYP2C19	0.646 [0.566–0.738]	1.10E-10
Visual impairment (*n* = 543)	CYP2C19	0.646 [0.536–0.778]	4.31E-06
Asthenia (*n* = 1,158)	CYP2C19	0.643 [0.561–0.738]	2.90E-10
Anxiety (*n* = 1,683)	CYP2C19	0.638 [0.567–0.718]	1.10E-13
Hypotension (*n* = 745)	CYP2C19	0.611 [0.518–0.72]	4.12E-09
Diarrhoea (*n* = 1,554)	CYP2C19	0.601 [0.531–0.68]	8.00E-16
Pain in extremity (*n* = 608)	CYP2C19	0.583 [0.487–0.698]	4.19E-09
Erectile-dysfunction (*n* = 331)	CYP2C19	0.56 [0.442–0.71]	1.70E-06
Loss of consciousness (*n* = 650)	CYP2C19	0.549 [0.461–0.655]	2.74E-11
Dyspnoea (*n* = 1,300)	CYP2C19	0.54 [0.472–0.617]	1.84E-19
Premature baby (*n* = 198)	CYP2C19	0.505 [0.371–0.686]	1.23E-05
Hypertension (*n* = 643)	CYP2C19	0.502 [0.420–0.601]	5.56E-14
Sleep apnoea syndrome (*n* = 463)	CYP2C19	0.473 [0.384–0.584]	2.51E-12
Pain (*n* = 934)	CYP2C19	0.468 [0.400–0.546]	8.85E-22
Seizure (*n* = 669)	CYP2C19	0.429 [0.358–0.514]	5.48E-20
Drug interaction (*n* = 927)	CYP2C19	0.412 [0.35–0.48]	1.14E-27
Fall (*n* = 1,316)	CYP2C19	0.404 [0.352–0.464]	2.54E-37
Serotonin syndrome (*n* = 377)	CYP2C19	0.38 [0.299–0.483]	2.62E-15
Pyrexia (*n* = 726)	CYP2C19	0.367 [0.306–0.44]	1.40E-27
Hyponatraemia (*n* = 808)	CYP2C19	0.312 [0.261–0.373]	2.61E-37
Small for dates baby (*n* = 306)	CYP2C19	0.186 [0.135–0.258]	2.52E-24
Electrocardiogram-QT-prolonged (*n* = 351)	CYP2C19	0.175 [0.129–0.239]	2.88E-28

## Discussion

This study considered that genes encoding the CYP2C19 and CYP2D6 drug metabolism enzymes are expressed at various quantifiable levels within anatomic organs and hypothesized that ADRs were associated with altered physiology in those organs. The results confirmed the hypothesis that SSRI substrates of CYP2C19 are associated with the following pregnancy-reproduction ADRs: foetal exposure during pregnancy, premature baby, and small for dates baby. One paradox to the pregnancy-reproductive-CYP2C19 results is that the uterus has the second lowest gene expression for CYP2C19 of all the 53 non-diseased anatomical structures as measured by RNA-Sequencing and reported in GTEx. Further, though not directly inferred, the GTEx results showing the suprapubic skin tissue as the sixth ranking anatomical structure expressing the CYP2C19 gene may relate to both pregnancy-reproductive ADRs as well as erectile-dysfunction, as illustrated in Table 1 and Fig. 3.

Further, the study results suggest that CYP2C19 SSRI substrates are associated with gastrointestinal absorption ADRs: diarrhoea, hyponatraemia, and again small for dates baby as well as pain-related ADRs: chest pain, pain in extremity, and pain. These results are informative due to most pain relieving opioids are metabolized by CYP2D6 and no pain-related ADRs showed increased odds with SSRIs metabolized by CYP2D6 (Zanger & Schwab, 2013). To continue, the results also suggest that CYP2C19 SSRI substrates are significantly associated with coordination and the respiratory system ADRs: dyskinesia, fall, dyspnea, sleep apnoea syndrome. Lastly, CYP2C19-related SSRI ADRs were associated with physiological modulation of the autonomic nervous system: seizure, hypertension, hypotension, QT prolongation, and serotonin syndrome, as shown in Table 1. However, this generalization is not absolute due to hyperhidrosis and tremor showing bias toward CYP2D6.

Existentially, SSRIs inhibit the function of the presynaptic serotonin transporter (SERT) that is encoded by the solute carrier family 6A4 (SLC6A4) gene (Stelzer et al., 2016). Among the human anatomical tissues expressing the SLC6A4 gene, the top three organs are the lung (*n* = 427, median TPM = 36.38), small intestine terminal ileum (*n* = 137, median TPM = 7.8), and the esophageal mucosa (*n* = 407, median TPM = 1.770) (GTEx Consortium, 2017). The brain region with highest gene expression for the SERT is the cerebellar hemisphere (*n* = 136, median TPM = 0.48) and is ranked 11th among 53 tissue sites assessed using RNA sequencing in GTEx (GTEx Consortium, 2017). Therefore, the study results reporting dyspnea and sleep apnoea syndrome may be best explained by the lung having the highest tissue-specific gene expression for the SERT. The pregnancy associated ADRs shown in Fig. 3 and Table 1 are likely also attributed to the SLC6A4 expression level in the small intestine terminal ileum, among other mechanisms are associated with absorption in CYP2C19 RNA abundance in the gastrointestinal systems mentioned above.

Published studies corroborating increased odds of QT prolongation among CYP2C19 SSRI substrates suggests a rationale of the sertraline inhibiting cardiac channels and both citalopram and escitalopram blocking the human ether-à-go-go-related gene currents (Chae et al., 2014; Lee et al., 2012; Witchel et al., 2002). There is much evidence to suggest that a decrease in plasma serotonin, as done by SSRIs, are associated with improved clinical outcomes. A study conducted at the Mayo Clinic measured plasma serotonin levels at baseline, prior to initiating citalopram and escitalopram treatment, at week 4, and 8-weeks in patients diagnosed with major depressive disorder (*n* = 306), found that serotonin blood levels decreased over time (*p* < 0.001) and were associated in improved clinical outcomes (Gupta et al., 2016). These results are informative due to citalopram and escitalopram are both CYP2C19 substrates and that serotonin may have a role in ventricular repolarization leading to a prolonged QT-interval as suggested by recent study in mice (Cui et al., 2018).

Another study conducted in three psychiatric hospitals in Israel reported that lower plasma serotonin levels were evident in healthy controls (5.91 ± 6.74 ng/ml) vs. suicidal inpatients (10.90 ± 9.74), non-suicidal inpatients (13.87 ± 10.28), and emergency room subjects (11.89 ± 10.78), *p* < 0.001 (Tyano et al., 2006). For comparative purposes, octreotide decreases plasma serotonin and is indicated in patients with a tumor that secretes serotonin, which commonly occurs in the *terminal ileum*, and known as a carcinoid tumor (O’Toole et al., 2000). Despite SSRIs and octreotide having different mechanisms by both drugs result in a decrease in plasma serotonin levels and improved symptoms clinical (Holck et al., 2019; O’Toole et al., 2000).

In reference to the CYP2D6-biased ADRs, as hypothesized, SSRIs metabolized by CYP2D6 were significantly associated with cognitive processes and symptoms seen in altered mental status: nightmare, sleep disorder, paranoia, mood swings, withdrawal syndrome, hallucination, confusional state, suicidal ideation, and memory impairment, as shown in Table 1 and Fig. 3. These results suggest involvement of the thyroid, cerebellum, and pituitary gland in altered mental status in humans as suggested by the transcript abundance from GTEx and shown in both Figs. 2 and 3 (Nordholm et al., 2013; Pariante et al., 2004). Its is well established that hyperhidrosis, as found ranked 5th in CYP2D6 transcript abundance, is often seen in patients suffering from acromegaly (Levy, 2004). Similarly, tremor, a CYP2D6-biased ADR, is seen in patients experiencing thyroid dysfunction and the GTEx results, shown in Fig. 3, illustrates that the thyroid is ranked 3rd among all organs in CYP2D6 gene expression.

Restful sleep is an important factor in treating patients in psychiatry. A recent pharmacogenomic randomized single-blind clinical trial evaluating the clinical utility of pharmacogenomic-based prescribing vs. the treatment-as-usual of antidepressants in patients diagnosed with major depressive disorder, reported that sleep disturbance as the most commonly reported adverse event among both treatment groups (Han et al., 2018). In this study, patients treated with fluoxetine, fluvoxamine, and paroxetine—all CYP2D6 substrates—had a 337% increase in odds of nightmares and a 119% increased odds of sleep disorder and thus rejects the null hypothesis and affirms the association of the CYP2D6 SSRI substrates with of de-realization of thought processes.

The main study limitation is that some SSRI antidepressants require more than one metabolic pathway for biotransformation into metabolites. There is even recent evidence, from a genome-wide association study, that Tetraspanin 5 (TSPAN5) and Glutamaterich 3 (ERICH3) genes are associated with serotonin biosynthesis and metabolism (Gupta et al., 2016). However, as previously mentioned the SSRIs were grouped according to CYPs established by the U.S. FDA Table of Pharmacogenomic Biomarkers, clinical pharmacogenomic guidelines, and the Consensus Guidelines for Therapeutic Drug Monitoring in Neuropsychopharmacology which are major study strengths (Bank et al., 2018; Hefner, 2018; Hicks et al., 2016; U.S. Food & Drug Administration, 2016). Another major study strength is that the data analyzed in this study are based on real-world patient ADR cases reported to the U.S. FDA coupled with an inquisitive observation made from the NIH sponsored GTEx database.

## Conclusions

The results suggest that SSRIs metabolized by CYP2C19 have ADRs significantly associated with gastrointestinal absorption, pain, pregnancy-reproduction, and physiological modulation of the autonomic nervous system including seizures and serotonin syndrome. Whereas, SSRIs that are substrates of CYP2D6 have ADRs related to cognition that range from nightmares to memory impairment. In this unique methodology of grouping SSRIs based on well-established drug metabolizing pathways, this study provides physiological insight for further hypothesis testing of other classes of medications and may guide drug selection in psychopharmacology.

## Additional Information and Declarations

### Data Availability

The following information was supplied regarding data availability: Data analyzed in this study are available from the FDA Adverse Event Reporting System (FAERS): Latest Quarterly Data Files at: https://www.fda.gov/drugs/fda-adverse-event-reporting-system-faers/fda-adverse-event-reporting-system-faers-latest-quarterly-data-files.
